# Access to Medicines via Non-Pharmacy Outlets in European Countries—A Review of Regulations and the Influence on the Self-Medication Phenomenon

**DOI:** 10.3390/healthcare9020123

**Published:** 2021-01-26

**Authors:** Patrycja Oleszkiewicz, Jerzy Krysinski, Urszula Religioni, Piotr Merks

**Affiliations:** 1 Department of Pharmaceutical Technology, Faculty of Pharmacy, Collegium Medicum in Bydgoszcz, 85-089 Bydgoszcz, Poland; oleszkiewiczpm@gmail.com (P.O.); jerzy.krysinski@cm.umk.pl (J.K.); p.merks@uksw.edu.pl (P.M.); 2 Collegium of Business Administration, Warsaw School of Economics, Al. Niepodległości 162, 02-554 Warsaw, Poland; 3 Collegium Medicum, Faculty of Medicine, Cardinal Stefan Wyszyński University in Warsaw, Auditorium Maximum, bldg. 21, Room 201 (II Floor), st. Kazimierza Wóycickiego 1/3, 01-938 Warsaw, Poland

**Keywords:** non-pharmacy trade, pharmaceutical market, OTC medicines, pharmaceutical care

## Abstract

Non-pharmacy trade concerns the sale of medicinal products outside of pharmacies, such as limited-service pharmacies, supermarkets, petrol stations, shops open to the public, and kiosks. Access to medicinal products via non-pharmacy outlets varies across the European countries, with a general deregulation of this market area observable. Increasing the availability of medicines by allowing patients to obtain them outside of pharmacies contributes to the spread of self-medication. The aim of this article was to review the legal regulations enabling the non-pharmacy trade in OTC (over the counter) medicinal products in European countries, with particular emphasis on the analysis of active substances contained in medicines available in the non-pharmacy trade. This analysis has made it possible to distinguish three categories of countries: (1) where there is a non-pharmacy trade in OTC medicinal products, (2) where there is a limited non-pharmacy trade in OTC medicines, (3) where there is only a pharmacy trade in OTC medicinal products. In the context of these considerations, we highlight the impact of patient access to medicinal products via non-pharmacy sources on raising the prevalence of self-medication. This article identifies the advantages and risks of self-medication, emphasising the role of the pharmacist as an advisor to patients within the scope of the therapies used.

## 1. Background

With economic development in Europe and worldwide, a trend towards liberalisation of the trade in medicinal products, such as the non-pharmacy trade and non-pharmacy outlets development, has been observed [1,2]. The non-pharmacy outlets are defined as non-registered points of dispensing over the counter (OTC) medicines, for which there is no need to obtain a license for distribution and no need to fulfil inspection requirement for safe drug storage. The non-pharmacy outlets include supermarkets, petrol stations, drugstores, shops open to the public, and kiosks [3,4].

The countries across Europe determine the structure of their non-pharmacy trade through individual legal regulations. A pharmacy monopoly had been in force in many of the countries [5], but in recent years, a trend has been observed towards easing of the existing rules rather than tightening them [1,6,7]. Looking at the organisation of the non-pharmacy trade as a criterion, we can divide European countries into three categories:

1. a non-pharmacy trade exists and is regulated by law and concerns selected active substances;
2. no non-pharmacy trade;
3. there is only a non-pharmacy trade in preparations with a low therapeutic effect (e.g., herbal medicines), or is under the supervision of a pharmacist or other person qualified in pharmaceutical sciences.

The aim of the article was to analyse the active substances contained in OTC (over the counter) medicines that are available in the non-pharmacy trade in the European countries. This article also considers the benefits and risks of patient self-medication resulting from the increasing availability of OTC medicines to patients.

## 2. Material and Methods

The study involved 30 European countries’ medicines. The legal acts in force in individual countries were analysed.

We found legal acts on the websites of the Ministries of Health of individual countries. We also searched the scientific literature for articles describing the topic under study. On this basis, we distinguished 3 categories of countries: with a developed a non-pharmacy trade, without a non-pharmacy trade, and with a limited non-pharmacy trade.

Limited-service pharmacies were considered to be places where it is not possible to consult with qualified staff when making a purchase (hence the analysis of interactions and side effects excludes medicines available in limited-service pharmacies and facilities employing pharmacists).

## 3. Results

### 3.1. Pharmacy-Only Medication Trade

Thirteen countries among the analysed European countries have a monopoly on pharmacies for the sale of medicines.

In Austria, OTC medicines, including herbal and homeopathic medicines (registered as drugs due to their intended application), can only be sold in pharmacies [8].

In Belgium, only para-pharmaceuticals (toothpastes, dermocosmetics, food for new-borns and young children, vitamins, shampoos) can be sold outside of pharmacies or over the Internet [9].

In Finland, medicines can only be purchased in pharmacies, with the exception of nicotine replacement therapy products, which have been available outside of pharmacies since 2006 [10].

In France, medicines can only be purchased in pharmacies. Self-service pharmacies, where medicinal products are on shelves available to patients, are very popular. The consumer chooses the medication on their own, but their purchase is still consulted with the pharmacist [11].

In Greece, only food supplements and herbal medicines can be found in supermarkets and grocery stores [12].

Spain also conducts a limited non-pharmacy trade—medicines are only available in pharmacies. Herbal preparations can be purchased outside of pharmacies, provided that they do not contain any information about medicinal or prophylactic properties—they are not classified as medicines. [13].

Lithuania, Latvia, Estonia, Slovakia, Luxembourg, Cyprus, and Malta have similar drug policies. Only food supplements and medical devices are available outside of pharmacies in these countries [13].

### 3.2. Limited Trade in Medicinal Products Outside of Pharmacies

In some EU countries, a small range of medicines are made available for retail sale outside of pharmacies under certain conditions but without strict market regulation, including, for example, the list of drugs available outside of pharmacies. The main assumptions of this model in Bulgaria, Croatia, Germany, Portugal, Romania, and Switzerland are presented below.

#### 3.2.1. Bulgaria

In Bulgaria, medicines can only be sold in pharmacies, although some OTC products are available through vending machines operated by pharmacies, which can only be owned by pharmacists [14].

#### 3.2.2. Croatia

In Croatia, medicines are not available in supermarkets or grocery stores or at petrol stations—they must be provided by qualified personnel. Non-pharmacy trade of medicines from the OTC category is carried out in specialist shops where employment of a responsible person—a pharmacist—is obligatory. This person is responsible for purchase, collection, storage and dispensing of medicines, informing users about the method of administration, and precautions and possible side effects related to taking a given product. If a pharmacist is not employed full-time, it is necessary to hire another qualified person—a pharmaceutical technician [15]. These establishments trade only in a limited range of medicines. The list of substances that can be sold outside of pharmacies can be found on the website of the Croatian Agency for Medicinal Products and Medical Devices—HALMED [16].

For substances such as ibuprofen or paracetamol, maximum doses and limits have been established for package sizes that can be dispensed to patients outside of pharmacies. They concern both different forms of the medicines and the age of the person for whom the preparation is intended [17]. For example, medicines which contain the active substance—ibuprofen—may be marketed outside of pharmacies if the following conditions are met:

− oral administration:

- maximum acceptable dose—200 mg (application in adults and children over 12 years of age);
- maximum package size—20 capsules or tablets; 12 bags of granules.

− external administration:

- maximum permissible concentration: 10% (application in adults and children over 12 years of age);
- the package may contain a maximum of 5 g of ibuprofen [17].

Medicines containing ibuprofen, administered orally as a suspension or rectally as suppositories, are not approved for retail sale in specialist shops [17].

#### 3.2.3. Germany

In Germany, medicines containing disinfectants for external use only and oxygen are available outside of pharmacies [18].

#### 3.2.4. Portugal

The non-pharmacy trade of OTC medicinal products was introduced in Portugal in 2005. Only a pharmacist, a pharmaceutical technician, or a salesman under the supervision of a pharmacist may dispense medicines outside of a pharmacy, and only to persons who are over 16 years of age. The dispensing facilities must be registered with the national control authority, INFARMED (The National Authority on Medicine and Health Products, I.P.), in Portugal [19].

#### 3.2.5. Romania

Retail trade in medicines may only be carried out by pharmacies under the supervision of pharmacists and by non-pharmacy outlets (so-called druggist’s shops). These units are run by “asistenti de farmacie”—the Polish equivalent being a pharmaceutical technician, and only OTC medicines can be purchased there [20].

#### 3.2.6. Switzerland

In Switzerland, medicines are classified into five different categories depending on where they are dispensed and their availability (on/without a doctor’s prescription):

A—only on prescription in a pharmacy;
B—on the basis of a repeat prescription in the pharmacy;
C—without a prescription in the pharmacy;
D—without a prescription in a pharmacy or non-pharmacy outlets;
E—available in non-pharmacy outlets [21].

OTC medicines from the category D can be purchased both in pharmacies and in non-pharmacy outlets. In drugstores, medicinal products are administered by qualified personnel, where aside from the pharmacist, a so-called “drogisten”, who has undergone a four-year training in basic pharmaceutical knowledge, may be employed there [21].

In shops open to the public, unqualified personnel may only sell herbal medicines, a full list of which is available on the website of the Swiss Agency for Therapeutic Products [22].

In April 2017, a bill was presented in Switzerland which would introduce significant changes in the structure of the non-pharmacy trade in this country [23]. This law concerns the availability category C listed products—this list includes approximately 650 OTC products available without a prescription, but only after consultation with the pharmacist. These include analgesics such as ibuprofen in different doses, hydrocortisone as an ointment, doxylamine, dihydrocodeine in drops (in different doses), levonorgestrel, and ulipristal acetate (“the morning-after pill”). This list also includes many cold-related medicinal products such as combinations of ephedrine and codeine [23].

The bill provides for the elimination of category C. The bill provides for the elimination of category C, which would mean that once the reallocation is completed, these would be placed in category D and then moved to category E (sales permitted in all stores). The D-list includes, inter alia, analgesics such as low-dose paracetamol or xylometazoline nasal spray. The idea of moving away from the obligation to consult a pharmacist to the free sale of these medicines, for example in supermarkets, is to be assessed in terms of the risk to patients [23].

### 3.3. Countries with a Legally Regulated Non-Pharmacy Trade in Medicines from Established Lists

#### 3.3.1. The Czech Republic

In the Czech Republic, medication sales were restricted to pharmacies until the end of 1997. The sale of certain medicinal products (mainly herbal) outside of pharmacies has been allowed since 1998—at petrol stations or drugstores. The employees of non-pharmacy outlets have to undergo special training to ensure appropriate conditions for receiving, storing, and selling medicines [24].

Some OTC medicines such as ibuprofen at a maximum dose of 200 mg and paracetamol at a maximum dose of 500 mg may be purchased outside of pharmacies [25]. These products may be placed in self-service zones, however, most often they are separated from the rest of the products—on separate appropriately marked shelves. A complete list of non-pharmacy medicinal products is available in the Czech database of registered medicines.

#### 3.3.2. Denmark

In Denmark, shops outside the pharmacy sector can be authorised to sell OTC medicines. These preparations must be considered suitable for sale outside of pharmacies by the Danish Medicines Agency (belonging to the group of medicines issued according to categories HF, HX, or HX18). Over-the-counter veterinary medicines can also be sold—category HV [26].

Medicinal products in Denmark are divided into the following categories:

− HF—an unlimited number of packages can be purchased in non-pharmacy outlets;
− HX—outside of pharmacies, one can buy a maximum of one package, available only in small packages;
− HX18—HX, subject to the consumer being at least 18 years of age [27].

The purchase of medicines in non-pharmacy markets is also restricted by the age of the consumer—in addition to pharmacies, OTC medicines in dispensing categories HF, HX, and HV can be purchased by persons over 15 years of age. Drugs from group HX18 may only be purchased by patients over 18 years of age [27].

On the website of the Danish Medicines Agency is a list of OTC medicines that have been approved for use in non-pharmacy outlets. The list is updated daily. The list includes information such as the trade name of the medicinal product, its pharmaceutical form, and the dose and the category to which the medication belongs (HF, HX, HX18, or HV). The agents available on the list include non-steroidal anti-inflammatory medicines, antihistamines, topical anaesthetics, agents for hyperacidity, as well as sublingual tablets or nitroglycerin spray [28]. The employees in non-pharmacy outlets do not need pharmaceutical education [26].

Since 15 October 2006, stores have been allowed to obtain an authorisation to provide only drugs used for quitting smoking—nicotine replacement therapy preparations. Such stores do not offer the entire basic range of OTC medicines [26]. Approximately 3000 shops/companies in Denmark have the right to sell OTC products for people. About 300 shops are entitled to sell veterinary medicines [27]. In the case of certain OTC medicines, one package per patient per day is permitted by the Danish Medicines Agency. These medicines have been placed in the HX or HX18 dispensing category—with marketing authorisation for them outside of pharmacy [28].

Packages containing larger quantities of the active substance are placed in category HA or B, which means that they can only be sold in pharmacies by qualified personnel. For example, a 500 mg pack of paracetamol containing more than 10 tablets is classified as dispensing group HA18, while packages containing 10 tablets or fewer are classified as HX18. The range of OTC medicines belonging to the HX or HX18 category was updated in September 2013 [29].

#### 3.3.3. The Netherlands

Since the introduction of the Act on Medicines on 1 July 2007, three categories of OTC medicines have been defined: those issued only in a pharmacy (UA); those issued in a pharmacy or non-pharmacy outlets, the so-called “drogist” (category UAD); and medicines introduced to general sales (AV). Before July 2007, the third category did not exist [30].

The majority of OTC medicines belong to the UAD category, and in order to reduce the risk from their misuse and abuse, these products must be dispensed under the supervision of trained pharmacy or drugstore personnel [30].

In accordance with Article 4.2 of the 2007 Drug Control Regulation of the Minister of Health, the Dutch Medicines Evaluation Board—CBG (College ter Beoordeling van Geneesmiddelen) decided on the following criteria for classifying a medicine as AV:

− The active ingredient of an over-the-counter medicine available without a prescription has been granted a marketing authorisation in the Netherlands or in the United States for at least 5 years;
− the risk of health damage after taking the medicine is negligible;
− there is no evidence of misuse;
− the number of units in the package is relatively small;
− the packaging and leaflet warn of potentially dangerous situations [29].

Additionally, on 9 December 2011, a sixth criterion was added:

− the availability of verbal advice from a pharmacist or other qualified person is not required [30].

Examples of these medicinal products available in Danish non-pharmacy outlets are nicotine patches, analgesics (e.g., paracetamol or ibuprofen), hyperacidity drugs (e.g., ranitidine, antacids, proton pump inhibitor—esomeprazole), antihistamines (cetirizine, loratadine), and anti-diarrhoeal loperamide. A list of all drugs is available on the website of the College ter Beoordeling van Geneesmiddelen [31]. In the list, for some medicinal substances, maximum doses, and quantities of a given drug in a package are determined.

The applicable legal norms do not specify appropriate regulations governing where drugs should be placed at non-pharmacy outlets. In supermarkets, they are usually placed on shelves separated from other products and are also specially labelled as medicinal products.

#### 3.3.4. Ireland

In Ireland, the medicinal products which, in the opinion of the Health Products Regulatory Authority (HPRA), may be sold or supplied in a way other than by or under the supervision of a pharmacist and guarantee a reasonable level of safety in their use, are known as the “general sales list” medicines (GSL). They were classified in the category “supply through general sales” and thus marked in the search engine on the Health Products Regulatory Authority (HPRA) website [32].

The medicines available outside of pharmacies include preparations with paracetamol; expectorants with guaifenesin; antacid—containing magnesium, calcium or ranitidine salts; preparations for a sore throat; and agents with acetylsalicylic acid (no more than 24 tablets per package). Medicines containing ibuprofen cannot be purchased outside of pharmacies [32].

Special requirements have been defined for the sale of paracetamol outside of pharmacies. These regulations were introduced due to the prevalence of deliberate poisoning with this medication, often with fatal consequences, in Northern Ireland [33].

The sale of medicinal products from vending machines or any other mechanically or electronically controlled self-service device is prohibited in Ireland [34].

#### 3.3.5. Poland

Trade in medicinal products in Poland takes place in accordance with the rules laid down in law—the Act of 6 September 2001 Pharmaceutical law. Non-pharmacy outlets (excluding limited-service pharmacies) sell only OTC medicines with active substances from the list published by the Minister of Health [35].

According to the applicable legal standards, the sale of medicinal products may be conducted in pharmacies, pharmacy outlets, and non-pharmacy outlets, which may include herbal and medical shops; specialist medical supply shops; and public shops, e.g., grocery stores, roadside kiosks, and petrol stations, which are established on the principles set out in the Act on Business Activity. Herbal and medical shops, as the only ones among non-pharmacy outlets, must be operated by persons with appropriate qualifications, i.e., by a person with a master’s degree in pharmacy, a doctor, a nurse, a pharmaceutical technician, or a person with at least secondary education and knowledge acquired during a course in herbal medicine (used as medicinal products) [35].

Due to patient safety, only those medicines which meet strictly defined criteria have been admitted to the non-pharmacy trade, e.g., herbal medicinal products whose composition contains active substances authorised for trading in Poland in medicines issued in pharmacies without a prescription for a period of at least 5 years [35].

On the website of the Ministry of Health, there is a list of 52 active substances approved for marketing outside of pharmacies. It contains ingredients of drugs with both local and systemic effects. These include analgesics such as paracetamol; ibuprofen; naproxen; acetylsalicylic acid; preparations for sore throats containing lidocaine, choline salicylate, chlorchinaldol, benzalconium chloride, or flurbiprofen; neutralising drugs used for hyperacidity; preparations for gastrointestinal disorders; and nicotine replacement therapy [36].

There are no limits to the package size or strength of a given medication. There are no legal requirements concerning the place where medicines should be placed. They are usually placed at checkout counters, and in larger shops there are separate shelves, but they do not have to be marked or separated in any way from other products sold in a given facility.

#### 3.3.6. Slovenia

In Slovenia, OTC medicines can be distributed in pharmacies and specialist shops. Like in pharmacies, sale is possible only by an authorised person; the minimum required education for dispensing medicines in these facilities is a pharmaceutical technician who has passed a qualification test [37].

The medicines available in the non-pharmacy trade have low biological activity and a relatively narrow range of adverse effects. They are used to correct mild symptoms and health problems. They are considered relatively harmless by the marketing authorisation institution JAZMP (Javna agencija Republike Slovenije za zdravila in medicinske pripomočke) [38].

The place of medicines in the store must be physically separated from other products and labelled so that consumers can easily distinguish them; the medicines can only be sold in their original packaging. A microclimatic condition monitoring system must work [39]. It is obligatory to keep appropriate documentation in a way that allows the immediate withdrawal of medicines and possible complaints [40].

#### 3.3.7. Sweden

In Sweden, the sale of selected medicines from the OTC category has been possible outside of pharmacies in licensed facilities since 2009. Examples of places where these products are available are small 24/7 shops, supermarkets, grocery stores, kiosks, and petrol stations. The sales statistics must be registered and reported to the so-called “Apotekens Service” so that the trading is monitored [41].

Pharmaceuticals must be separated from other products in a way that allows the staff to exercise visual supervision over them at all times during shopping ours. It is preferable to place medicinal products in closed cabinets or behind the counter, or, after obtaining the appropriate authorisation, also in a vending machine [40]. Medicines without a prescription may not be sold outside of pharmacies to persons under 18 years of age. Medicinal OTC products, with the exception of nicotine replacement therapy preparations, may not be sold in commercial premises which have been granted permission to sell alcoholic products [41].

The list of medicines marketed outside of pharmacies is available on the website of the Swedish Medical Products Agency. The list includes medicines ranked according to their use, including trade name, pharmaceutical form, dose, and maximum quantity in the package [42]. It contains medicines for hyperacidity and indigestion containing aluminium hydroxide, calcium carbonate, magnesium carbonate, magnesium hydroxide, and ranitidine. The list includes medicinal products for constipation such as lactulose or macrogol, as well as for bloating, containing dimethicone and simethicone. Antifungal medicines include terbinafine and ketoconazole in ointments. Bromhexine and lozenges for a sore throat, which include amylmetacresol or dichlorobenzyl alcohol, are also available outside of pharmacies. Anti-histamines containing such substances as cetirizine, loratadine, and desloratadine are also available. The list also includes analgesics, anti-inflammatory, and antipyretic drugs such as ibuprofen, naproxen, and acetylsalicylic acid [42].

As from 1 November 2015, paracetamol tablets are no longer permitted to be sold in non-pharmacy outlets in Sweden. This decision was taken in April 2015 by the Swedish Medical Products Agency (MPA) and applies to tablets intended to be ingested in whole. Other forms of paracetamol such as syrup or effervescent tablets are not affected by this decision. The regulation was approved on the basis of the increase in paracetamol poisoning, where it was taken with the intention of causing health damage. The availability of paracetamol in the form of tablets outside of pharmacies was considered a risk factor for drug abuse [43].

In Sweden, contraceptives containing levonorgestrel and the “morning-after pill” ellaOne containing ulipristal acetate are also available in non-pharmacy outlets. It is also possible to buy preparations with estriol and herbal medicines used to treat symptoms of benign prostatic hyperplasia or to reduce menopause-related ailments [44].

#### 3.3.8. Hungary

In 2007, the Hungarian Minister of Health published a list of OTC medicines that may be sold in retail facilities other than pharmacies, e.g., supermarkets, pharmacies, and petrol stations.

The list of OTC medicines includes analgesics (ibuprofen, acetylsalicylic acid, paracetamol), flu medicines (phenylephrine, pheniramine, guaifenesin), antifungal creams, medical disinfectants, anti-inflammatory agents, antihistamines, gastric acid neutralisation medicines, vitamins, certain ointments, eye drops, and nasal sprays. This list is regularly updated [44].

Apart from pharmacies, medicinal products may be sold only to persons over 14 years of age. The store must be licensed and a space or a separate section of the room must be provided, which should clearly indicate that medicines are sold in these particular areas. These products shall be stored in closed cabinets. With the exception of medical devices, herbal products, or teas, medicines should be positioned in such a way that consumers do not have direct access to them [45].

The following have been approved outside of pharmacies in Hungary:

− gastric acid neutralisation medicines (aluminium hydroxide, magnesium hydroxide);
− medicines used in functional disorders of the intestines (simethicone, diosmectite);
− osmotically active laxatives (lactulose, macrogol);
− medicines containing adsorbent agents such as activated carbon;
− preparations containing calcium carbonate;
− preparations containing magnesium citrate;
− local agents against haemorrhoids;
− topical agents for sealing capillaries;
− antifungal preparations for topical use;
topical antihistamine preparations against itching (dimetindene);
disinfectant preparations containing iodine;
− disinfectant tablets containing chlorine and hydrogen peroxide;
− preparations against warts and blisters;
− preparations containing ibuprofen:
○ no more than 400 mg of active substance and up to 10 units per package;
○ suspensions containing ibuprofen;
○ suppositories containing ibuprofen, maximum 5 per pack;
− preparations for topical use for muscle and joint pain containing ibuprofen or salicylic acid derivatives;
− painkillers and fever relievers for adults, containing acetylsalicylic acid for oral administration, up to a maximum of 500 mg and up to 12 units per package;
− preparations containing paracetamol:
○ tablets up to 500 mg, maximum six tablets per pack;
○ syrups containing paracetamol;
○ suppositories containing 125 mg, 250 mg, or 500 mg may be dispensed in packages containing a maximum of six suppositories;
− preparations with melatonin, containing a maximum dose of 3 milligrams;
lozenges for a sore throat (benzydamine, cetylpyridine, lidocaine, amylmetacresol, dichlorobenzyl alcohol, and others);
− expectorants containing essential oil or plant extract;
− xylometazoline in the form of drops or nasal spray, nasal spray, up to 0.05% of medication concentration;
− sodium cromoglicate, 2% solution, the largest pack may contain 10 mL of product, in case of multi-dose form not more than 3 mg/dose;
− complex homeopathic drugs [46].

#### 3.3.9. The United Kingdom

In the UK, consumers can purchase medicines from retail outlets such as corner shops, supermarkets, kiosks, and petrol stations.

There are three categories of medicines:

1. POM—prescription-only medicine, medicines available only by prescription;
2. P—pharmacy-supervised sale medicine, these medicines must be dispensed under the supervision of a pharmacist;
3. GSL—general sales list medicine, medicines are available both in pharmacies and in non-pharmacy outlets.

The release of GSL medicines requires that the following conditions are met:

− the place where the medicinal product is sold, offered, put up for sale, or to which the medication is ordered, must be premises owned by the operator and only if it is possible to close the premises to consumers;
− sales from vending machines with medicines may be conducted only from machines located in spaces with the possibility of being closed to unauthorised persons;
− the medicinal product must be delivered for sale in its original packaging and may not be opened until it is administered [47].

Medicinal products available outside of pharmacies are accepted for common easily recognisable ailments, which usually last about 2–3 days. They cause few adverse effects during normal use.

Classification into a given availability category is part of the marketing authorisation proposed by the manufacturer of the product. Due to a lack of experience in its use, a new medication is automatically classified as a POM. If used by a large number of patients, it can be reclassified as P. This means that it is considered safe enough to be dispensed to a patient without medical supervision. In the same way it is possible to change the category of a medicine to GSL.

The reclassification of medicines is an ongoing process in the UK. Until 2005, the country was considered the world leader in making changes in the status of medicines from other groups to GSL. As an example, the reclassification of a Nexium Control preparation containing a maximum of 20 milligrams of the active substance esomeprazole, approved in 2015 by the Medicines and Healthcare products Regulatory Agency (MHRA), from category P to GSL, could be cited. Two packs containing 7 or 14 tablets have been approved for non-pharmacy sale. This is the first proton pump inhibitor to be transferred to the list of GSL medicines on sale in the UK [48].

Other types of GSL medicines used to treat hyperacidity-related symptoms are also available on the market. These include gastric acid neutralisers, e.g., calcium carbonate or magnesium hydroxide, alginates (they form a mechanical barrier on the gastric mucosa surface and thus reduce reflux), and H_2_ histamine receptor antagonists—ranitidine and famotidine [49].

To reduce the risk of damage caused by improper use, many drugs intended for sale outside of pharmacies contain reduced doses in a single package. As a rule, there is also information to get help from a professional if the health condition does not improve or if it deteriorates. The same medication for sale outside of a pharmacy may be recommended for limited treatment, and when dispensed in a pharmacy, is used for a wider range of indications. Products dispensed by a pharmacist may be used in higher doses and for longer periods of time than medicines in general sales, which cannot also be advised to children or pregnant women.

The Medicines and Healthcare Products Regulatory Agency’s website contains lists with detailed information on the conditions for dispensing medicinal products. List B includes a standardised list of authorised substances in non-pharmacy outlets—GSL. The lists are updated following new reclassifications. Details of new reclassifications are provided in List C, with an appropriate reference to Lists A or B [50].

In order for a medicinal product containing active substances such as aloxiprin (acetylsalicylate aluminium—polymer of aluminium oxide and acetylsalicylic acid), acetylsalicylic acid (ASA), or paracetamol (or any combination of these substances) to be made available for sale outside of a pharmacy, it must meet the conditions set out in the Human Medicines Regulations of 2012. A pharmacy marketing authorisation is possible only if the drugs are in separate and individual packages, containing not more than a precisely defined amount of product [51].

In the UK, there are no requirements for a mandatory range of medicines that must be available in a non-pharmacy outlet. The marketing unit concerned is free to choose the medicinal products in the GSL category that will be made available. The law does not require the provision of statistics on the trade of medicines from non-pharmacy outlets, nor is there a specific place where medicinal products should be placed in stores.

#### 3.3.10. Italy

In 2006, all OTC medicines were authorised for sale outside of pharmacies. The sale of those products may take place in pharmacies, in drugstores called “parafarmacie” in Italy and in designated areas called “health corners” in supermarkets. OTC medicines are placed on shelves accessible to the public or behind the counter. Only a pharmacist can sell them. Price promotions for medicines are prohibited [52].

#### 3.3.11. Norway

OTC medicines in Norway can be sold in pharmacies and in licensed “medicine outlets”, which are branches of a local pharmacy. The pharmacy owner is responsible for the training of staff [53].

A small number of OTC medicinal products containing active substances approved by The Norwegian Medicines Agency have been approved for sale at petrol stations, supermarkets, kiosks, and grocery stores. These medicines can also be purchased from vending machines [54].

A list of authorised non-pharmacy medicinal products is published on the website of The Norwegian Medicines Agency, together with the requirements for their storage and dispensing. This list is in the form of a table which distinguishes, for a given active substance: its dosage, pharmaceutical form, and restrictions on the maximum number of units in one package. The agents, which are marked yellow in this list, must be stored in a way that prevents self-service, i.e., in a closed cabinet or behind the counter. These are preparations containing acyclovir, ibuprofen, diclofenac, levonorgestrel, meclozine, oxymetazoline, paracetamol, penciclovir, ulipristal acetate, xylometazoline, and its combination with ipratropium [54].

It is mandatory to clearly distinguish between medicines and other products and to place them in a way that allows supervision of personnel. They cannot be easily accessible to minors, and it is assumed that they should be at least 140 cm from the floor [53].

Every non-pharmacy marketing establishment in Norway must have a minimum range of medicinal products prescribed by law, after obtaining approval to operate them.

Only persons over the age of 18 may purchase medicines in a place other than a pharmacy. For some substances, there are restrictions on their sale. Examples are preparations containing paracetamol or nasal topicals with xylometazoline, oxymetazoline, or ipratropium bromide. Only one package containing the same active ingredient per consumer may be dispensed. This means that a person cannot buy both a tablet and a suppository containing paracetamol (as the only ingredient or one of several). An exception to this rule is the simultaneous purchase of preparations for adults and children. A single sale of other forms of a medication containing the same active substance in different doses is considered legal [53].

It is forbidden for shop staff to give advice on the choice of a medication or information on its properties and use. If advice is required, the employee must refer the person to a pharmacist or doctor. It is also prohibited to advertise medicinal products inside or near a shop. Discounts, promotions on medicines, or other means to promote sales are not permitted [53].

## 4. Discussion

Our analysis indicated that out of the 30 European countries, patients in 10 countries may purchase medicines from trading points other than a pharmacy or a limited-service pharmacy specified in lists agreed by the Ministry of Health of the country concerned, called “general sales lists”. They include Poland, Ireland, the Netherlands, Slovenia, Hungary, Italy, the Czech Republic, Denmark, and Sweden. Among the analysed countries where non-pharmacy trading is allowed, Norway also permits non-pharmacy trading. In Italy and Slovenia, it is possible to purchase medicines outside of pharmacies, but sales are conducted by a pharmacist.

The European countries where only pharmacies have a monopoly on dispensing medicines are Austria, Belgium, Cyprus, Estonia, Finland, France, Greece, Lithuania, Luxembourg, Latvia, Malta, Slovakia, and Spain. In addition to pharmacies, a limited range of medicines in limited-service pharmacies are available in the following countries: Bulgaria, Croatia, Germany, Portugal, Romania, and Switzerland (Table 1).

One of the consequences of the pharmaceutical market deregulation may be the increase in the popularity of self-medication. According to the World Health Organization, self-treatment is the use of medicines by the consumer to treat diseases or symptoms diagnosed by themselves [55]. This phenomenon refers to OTC medicinal products as well as those prescribed by a physician, and also to changes in the recommended dosage. In practice, this term also includes reciprocal treatment by family members or friends, especially when a child is being treated.

The development of self-treatment is associated with raising the level of patient knowledge, and also with the promotion of healthy lifestyles in the media; widespread advertising of medicines; and largely increasing their availability through their presence in public shops, supermarkets, or kiosks [56,57,58]. The self-administration of medicines is also supported by limited access to healthcare or long waiting times for a doctor’s appointment [59].

The prevalence of self-medication leads to a decrease in the share of healthcare in the treatment of mild disorders and has a positive impact on the quality of life of patients suffering from chronic and recurrent diseases. Examples where self-treatment can be implemented include a cold or flu, digestive disorders (including heartburn), and mild to moderate pains such as headaches and muscle pains [60,61,62].

Self-medication contributes to increasing patient knowledge. It raises a sense of responsibility for one’s own health and encourages active prophylaxis. When implemented consciously and responsibly, it leads to an improvement in quality of life, which in turn has a positive effect on the health of society. The introduction of self-treatment generates savings for the state budget (annual savings from the transfer of 5% of prescription medicines to the OTC category in seven European countries were estimated at over EUR 16 billion [63]), and also time savings, both for patients and the entire healthcare system. This contributes to an improvement in the quality of services provided by general practitioners and to the extension of the scope and level of professional care for seriously ill people [64].

Many medicines from the OTC category, such as those in the non-pharmacy market, may lead to the development of addictions. There are cases of misinterpretations of pain or other ailments that create a situation in which an ever-increasing dose of drugs is consumed, which in turn can result in poisoning.

However, the benefits of patients practising treatment on their own depend on whether they are taking it in a rational and responsible manner. The format in which the information about the medication contained in the package leaflet is provided is not without significance [58]. Legal regulations implemented by the European Union concerning its form are aimed at ensuring that safety standards are met. According to the additional Directive of 2004 (Directive 2004/27/EC of the European Parliament and of the Council of 31 March 2004), manufacturers of OTC medicines are obliged to display information in braille on the packaging, as well as to ensure that conditions are in place to allow the visually impaired and blind to access information about the medication [65].

However, studies indicate negative consequences related to the lack of familiarity of patients with the leaflet attached to the medicines [60]. One of the most serious threats of self-treatment is polypharmacy, involving the patient taking multiple medicines simultaneously. The polypharmacy risk group includes in particular elderly patients, who are often treated with several different medicinal products [66]. It is estimated that almost 50% of elderly people take one or more medicines, which are necessary for medical reasons [67]. A prospective cohort study among hospitalised elderly patients showed that the probability of interaction increases with the number of agents used. In patients taking from five to nine different medicines, the probability was 50%, and the risk increased to 100% when 20 or more medicines were used. Thus, polypharmacy is a phenomenon containing the predisposition to interaction [67].

Interaction is a phenomenon where two or more pharmacological agents taken at the same time interact with each other and result in modification of the activity of some of them. Interactions are estimated to cause 5–20% of all adverse reactions. About 30% of medication complications resulting in patient’s death are considered to be the consequences of interactions [68].

Currently applied pharmacotherapies are usually associated with the simultaneous use of many medicines. The susceptibility to self-treatment, use of OTC medicines under the influence of advertising, older age of the patient, or related polypharmacy, as well as the coexistence of many different diseases, are factors favouring the occurrence of harmful drug interactions [66].

In this context, the role of pharmacists should be emphasised as the main source of information on therapy for patients. Properly conducted pharmaceutical care can contribute to the development of self-treatment and increase patient awareness of the benefits and risks (Table 2). Trust in pharmacists helps to change the inappropriate habits of patients, increasing the likelihood of compliance with the recommendations for the introduced pharmacotherapy. Pharmaceutical care is becoming an indispensable element of the modern healthcare system [69,70,71].

Self-medication conducted in a reasonable and controlled manner has positive effects. With the implementation of appropriate pharmaceutical care, it can take on a rational dimension. It is also necessary to provide pro-health education for the patient on how to administer medicines and lead a healthy lifestyle on their own. Research monitoring the effects of medication therapies used by patients is also an important aspect.

### Limitations

To the best of our knowledge, in some countries, non-pharmacy outlets require pharmacists to be present. For example, in the UK, pharmacists must be present to provide OTC medicines labelled as group P, even outside pharmacists. Due to the lack of available literature on this subject, we could not accurately compare the situation between countries. However, it should be underlined that the presence of a pharmacist may significantly affect the safety of taking OTC medicines among patients.

## 5. Conclusions

The regulation of non-pharmacy marketing of medicinal products in the analysed countries varies. In Europe, two directions of change concerning the non-pharmacy market for medicinal products can be observed. In countries where this market has existed for a long time, regulations are introduced to control it more closely, while in countries where the sale of medicines outside of pharmacies is prohibited, bills are drafted to allow it.

Of the analysed European countries with a non-pharmacy trade, the fewest non-pharmacy medicines are available in the Czech Republic and Ireland. Relatively liberal countries in this respect are the northern countries such as Sweden, Norway, and Denmark, as well as the United Kingdom. Poland stands out from other countries with no restrictions on maximum quantities or doses of medicines sold in outlets other than a pharmacy (this applies especially to non-steroidal inflammatory medicines or paracetamol).

Patient access to medicinal products translates into the prevalence of self-medication. Despite many positive effects of this phenomenon, the resulting risks, such as polypharmacy and drug interactions, should be emphasised. Additionally, OTC medicines can mask the symptoms of serious diseases, delaying the diagnosis of patients.

The important role of the pharmacist in the treatment process should be emphasised, which is to provide patients with reliable information on pharmacotherapy, especially on the use and effect of medicines and on possible interactions with both food and other medicines. Relieving the burden on healthcare through self-treatment, conducted in a reasonable and controlled manner, has positive effects. The implementation of appropriate pharmaceutical care will help to popularise self-treatment and to introduce its rational dimension.

Systematic educational measures are necessary in order to reduce the harmful effects of improper self-treatment and to raise public awareness of the dangers involved. The patient must be aware that OTC medication, even if purchased in a convenience store like any other commercially available pharmacist, has both therapeutic and toxic properties.

As comprehensive pharmaceutical care is not provided in many countries, patients have to increase their awareness of possible mistakes when using medicines, especially OTC medicines. For this purpose, information campaigns, TV spots, or leaflets and brochures, available, for example, in pharmacies or primary care physicians, can be helpful.

Actions at the level of regulation of non-pharmacy sales of medicinal products, such as restrictions on package sizes or maximum dosages, as well as placing medicines in appropriately marked places clearly separated from other goods, may significantly affect patient awareness of the risks associated with the consumption of medicines.

## Figures and Tables

**Table 1:** Comparison of availability of medicines outside of pharmacies in the European countries.

There Is Only a Pharmacy Trade in Medicinal Products	There Is a Non-Pharmacy Trade in Medicinal Products	There Is a Limited Non-Pharmacy Trade in Medicinal Products
Austria Belgium Cyprus Estonia Finland France Greece Spain Lithuania Luxembourg Latvia Malta Slovakia	The Czech Republic Denmark The Netherlands Ireland Norway Poland Slovenia Sweden Hungary Italy	Bulgaria Croatia Germany Portugal Romania Switzerland

**Table 2:** Advantages and risks of self-medication.

Advantages of Self-Medication	Risks of Self-Medication
− increasing knowledge of the disease, educating patients − a sense of responsibility for one’s own health − change in lifestyle, improvement of its quality, − convenience − reducing the number of patient visits to healthcare facilities − reducing the cost of maintaining these facilities − relieving the doctors − savings in public funds	− polypharmacy − harmful side effects and adverse effects − drug interactions − postponing a consultation with a doctor − delayed diagnosis in case of serious diseases or complications

## Data Availability

Not applicable.
